# A brief review: adipose-derived stem cells and their therapeutic potential in cardiovascular diseases

**DOI:** 10.1186/s13287-017-0585-3

**Published:** 2017-06-05

**Authors:** Teng Ma, Jiacheng Sun, Zhenao Zhao, Wei Lei, Yueqiu Chen, Xu Wang, Junjie Yang, Zhenya Shen

**Affiliations:** Department of Cardiovascular Surgery of the First Affiliated Hospital & Institute for Cardiovascular Science, Soochow University, No.899, Pinghai Road, Suzhou, 215006 China

**Keywords:** Adipose-derived stem cells, Stem cell transplantation, Cardiovascular diseases, Differentiation, Paracrine effect

## Abstract

Adipose-derived stem cells (ADSCs) are easily obtained and expanded, and have emerged as a novel source of adult stem cells for the treatment of cardiovascular diseases. These cells have been shown to have the capability of differentiating into cardiomyocytes, vascular smooth muscle cells, and endothelial cells. Furthermore, ADSCs secrete a series of paracrine factors to promote neovascularization, reduce apoptosis, and inhibit fibrosis, which contributes to cardiac regeneration. As a novel therapy in the regenerative field, ADSCs still face various limitations, such as low survival and engraftment. Thus, engineering and pharmacological studies have been conducted to solve these problems. Investigations have moved into phase I and II clinical trials examining the safety and efficacy of ADSCs in the setting of myocardial infarction. In this review, we discuss the differentiation and paracrine functions of ADSCs, the strategies promoting their therapeutic efficacy, and their clinical usage.

## Background

Cardiovascular diseases (CVDs) are the leading cause of morbidity and mortality worldwide [1]. Ischemic heart disease (IHD), specifically acute myocardial infarction (AMI), is the most common type of heart disease. The survival of patients with AMI has been substantially improved by advanced medical treatments and device-based therapies. The current medical therapies are capable of rescuing the remaining viable tissues within the damaged heart, while the problem is an inability to replace lost myocardium with regenerated cardiovascular cells [2]. Heart transplantation remains the only option for patients facing fatal heart failure, while the number of donors is limited. Stem cell transplantation is emerging as a promising strategy to regenerate the ischemic myocardium in such patients.

Stem cells, including embryonic stem cells (ESCs) and adult stem cells (ASCs), are cells capable of self-renewal and differentiation into a variety of phenotypes, and could be used for the treatment of heart failure [3, 4]. Recently, clinical trials using various ASCs for cardiac regeneration have been performed [5, 6]. Bone marrow-derived mesenchymal stem cells (BM-MSCs) were the first to be recognized and the most extensively studied for CVDs [3]. However, harvesting of BM-MSCs is rather invasive and painful, with potential morbidity and low yields [7]. In contrast, obtaining adipose-derived stem cells (ADSCs) increases yields and reduces the pain in a simple procedure. Adipose tissue also has a significantly higher stem cell density than bone marrow (5% versus 0.01%) [7].

ADSCs were first discovered and defined as mesenchymal stem cells (MSCs) isolated from processed lipoaspirate by Zuk and colleagues in 2001 [8]. These cells have the potential to differentiate into cardiovascular lineages, such as cardiomyocytes (CMs), endothelial cells (ECs), and vascular smooth muscle cells (VSMCs) [7]. Fan et al. demonstrated that ADSCs differentiate more quickly into ECs and possess a stronger proliferation ability than BM-MSCs [9]. Furthermore, Noël et al. observed a similar adipocyte differentiation ability of ADSCs compared to BM-MSCs, as well as less osteogenic and chondrogenic differentiation efficiency in ADSCs compared to BM-MSCs [10]. The paracrine effect of ADSCs appeared to play the major role in the treatment of IHDs. ADSCs could secrete various protective factors including vascular endothelial growth factor (VEGF), hepatocyte growth factor (HGF), insulin-like growth factor-1 (IGF-1), and a variety of microRNAs. Uysal et al. observed a similar VEGF level secreted by ADSCs and BM-MSCs [11], while Ikegame and colleagues indicated a greater production of VEGF and HGF by ADSCs than BM-MSCs [12]. Given these advantages, ADSCs may be better candidates for myocardial regeneration than bone marrow-derived stem cells.

However, stem cell-based transplantation therapy faces the problems of poor donor cell engraftment and survival in the ischemic heart. Because of the above limitation, researchers introduced several new strategies to enhance cell survival and retention. Increasing numbers of animal and clinical studies have focused on ADSCs and their therapeutic application in treating IHDs. In this review, we will focus on the regenerative role of ADSCs, the methods for therapeutic improvement, and their clinical application in CVD.

### Differentiation ability of ADSCs

**Fig. 1:**
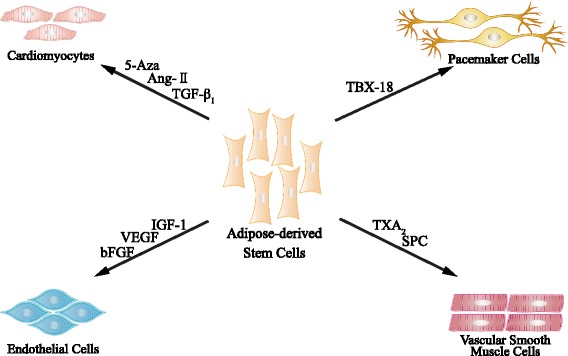
Differentiation ability of adipose-derived stem cells. ADSCs can differentiate into cardiovascular lineages, such as cardiomyocytes, endothelial cells, vascular smooth muscle cells, and pacemaker cells. Various reagents have been used for ADSC induction. *Ang* angiotensin, *Aza* azacytidine, *bFGF* basic fibroblast growth factor, *IGF* insulin-like growth factor, *SPC* sphingosylphosphorylcholine, *TBX-18* T-box 18, *TGF* transforming growth factor, *TXA*_*2*_ thromboxane A_2_, *VEGF* vascular endothelial growth factor

ADSCs play an important role in cardiovascular regeneration due to their ability to differentiate into a variety of cell lineages. Previous reports demonstrated that ADSCs were able to differentiate into CMs, ECs, and VSMCs, which are the main components of the cardiovascular system [13]. Various strategies of inducing ADSCs to differentiate into cardiovascular lineages were evaluated, such as application of biological reagents and genetic modification [2], which will be summarized in the following paragraphs (Fig. 1).

#### Induction of ADSCs into cardiomyocytes

ADSCs cultured in semi-solid methylcellulose medium spontaneously differentiated into cells with morphologic, molecular, and functional properties of CMs [14]. These CM-like cells expressed several cardiac-specific transcription factors, including GATA4, NKX2.5, MLC-2v, and MLC-2a, and displayed spontaneous and triggered action potentials. These beating clones comprised about 0.02% to 0.07% of CM-like cells at day 20. This was the first description of the spontaneous differentiation of ADSCs into functional CM-like cells.

Various reagents have been used to induce the differentiation of ADSCs into cardiomyocytes, i.e., 5-azacytidine (5-Aza), angiotensin II (Ang II) and transforming growth factor (TGF)-β1. 5-Aza, an s-triazine nucleoside analogue of cytidine, was the first agent used for cardiomyogenic differentiation of bone marrow stromal cells [15]. Based on its inductive potential, Rangappa et al. [16] applied 5-Aza to ADSCs and successfully induced the differentiation of ADSCs into cardiomyocytes in vitro. After stimulated with 5-Aza, 20–30% of cells showed binucleation and extended cytoplasmic processes with adjacent cells at 1 week. At 3 weeks, these cells began to beat spontaneously in culture. The differentiated ADSCs not only expressed cardiac-specific markers such as α-actinin and troponin-I, but also exhibited biological and electrophysiological characteristics of CMs [16]. Ang II regulates myocardial functions and has various bioeffects [17]. Song et al. induced ADSCs with Ang II to differentiate into CMs characterized by cardiac troponin-I and connexin-43 expression [18]. Although Ang II had a lower inductive efficiency compared to 5-Aza (18% vs. 21%), it was nontoxic to the cells and could be a good substitution for 5-Aza. Moreover, Gwak et al. used TGF-β1 to induce ADSCs to differentiate into CMs, as detected by immunofluorescence and flow cytometry [19].

Apart from these reagents, genetic modification of ADSCs has been used to induce their differentiation into cardiac pacemaker cells. *TBX18*, a T-box family member, is highly expressed in the progenitor cells of the sinoatrial node (SAN). Therefore, Yang et al. [20] transfected *TBX18* to ADSCs, and the modified cells were capable of differentiating into pacemaker-like cells. *TBX18* may enrich the efficiency of pacemaker-like cell differentiation by promoting the expression of pacemaker channel HCN4 [20].

#### Induction of ADSCs into endothelial cells

Endothelial dysfunction is common in conditions such as coronary artery disease, diabetes mellitus, and stroke [21, 22]. Stem cell transplantation-based therapeutic angiogenesis, including endothelial differentiation and paracrine effects, plays a key role in restoring endothelial function. The endothelial differentiation potential of ADSCs was first discovered in 2004 [23]. Several promising experiments indicated the differentiation capacity of ADSCs to ECs in vitro. Miranville et al. [23] demonstrated that a subset of ADSCs (CD34^+^/CD31^–^) was capable of differentiating into ECs when cultured in endothelial growth medium supplemented with IGF and VEGF. Under this condition, the cells showed a spindle-shaped morphology and high expression of EC markers such as CD31 and vWF. In contrast, Cao et al. [24] isolated another cell subset of ADSCs (CD34^–^/CD31^–^) and cultured them on matrigel supplemented with basic fibroblast growth factor (bFGF) and VEGF. The characteristics of this subset were in accordance with human umbilical vein endothelial cells. Moreover, Konno et al. [25] emphasized bFGF to be an effective inducer of EC differentiation with an induction rate of more than 85%. bFGF omission greatly diminished the ability of ADSCs to uptake Ac-LDL and downregulated EC marker expression, i.e., CD31, VE-Cadherin, vWF, VEGFR1, and eNOS. The in vivo differentiation potential of ADSCs into the endothelial lineage has also been demonstrated. Moon et al. [26] manifested the incorporation of ADSCs into the vasculature of mouse ischemic hindlimb and augmented capillary density after intravenous injection of the cells, which confirmed their EC differentiation capacity in vivo.

#### Induction of ADSCs into VSMCs

In addition to ECs and CMs, VSMCs are another important component of the cardiovascular system. In addition, VSMCs are critical for maintaining the physiological functions of the blood vessel wall. The characteristic markers of VSMCs are smooth muscle α-actin (SM α-actin), caldesmon, SM22, calponin, smooth muscle myosin heavy chain (SM-MHC), and smoothelin. Rodríguez et al. first reported the successful differentiation of ADSCs into SMCs in 2006 [27]. Cells were cultured in smooth muscle inductive medium consisting of MCDB131 and heparin for 6 weeks. These cells expressed VSMC markers and contracted in response to carbachol. Kim et al. [28] reported that U46619 (a thromboxane A2 analog) could induce differentiation of ADSCs to SMCs in vitro. ADSCs were treated with U46619 for 4 days, and these stimulated cells expressed VSMC-specific markers such as SM α-actin, calponin, SM-MHC, and smoothelin, and exhibited increased contractility thereby providing another option for the induction of ADSCs to VSMCs. Furthermore, Jeon et al. observed that sphingosylphosphorylcholine could induce the differentiation of ADSCs into VSMCs via modulation of the Smad2 pathway [29].

### Paracrine effects of ADSCs

**Fig. 2:**
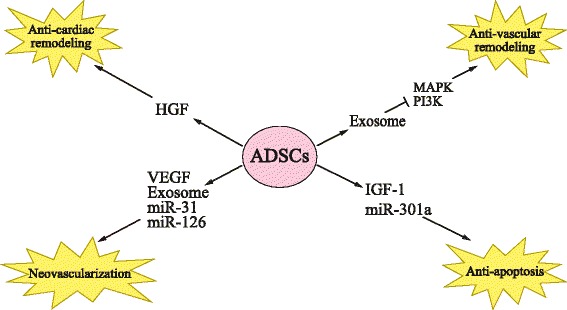
Paracrine effects of adipose-derived stem cells (*ADSCs*). ADSCs secrete vascular endothelial growth factor (*VEGF*), microRNA (*miR*)-31, miR-126, and exosomes for promoting neovascularization. The anti-apoptosis effect of ADSCs is mediated by insulin-like growth factor-1 (*IGF-1*) and miR-301a. The anti-cardiac remodeling effect is associated with hepatocyte growth factor (*HGF*). ADSC-derived exosomes inhibit mitogen-activated protein kinase (*MAPK*) and phosphatidylinositol-3-OH-kinase (*PI3K*) pathways, exerting an anti-vascular remodeling effect

Stem cell-based therapy shows great promise for regenerating damaged myocardium. ADSCs are currently the focus of considerable interest in the field of regenerative medicine and have been suggested as an ideal source for stem cell-based therapy. The recovery of cardiac function after cell transplantation is attributed to the paracrine effect rather than the direct differentiation of ADSCs [30]. In addition, ADSCs release different paracrine factors depending on the microenvironment. Under hypoxic condition, ADSCs secrete VEGF at a significantly higher level than under a normoxic condition [31]. Furthermore, ADSCs isolated from CAD or diabetic patients secrete a high level of HGF compared with healthy volunteers [32]. In this section, we summarize the paracrine effects of ADSCs (Fig. 2).

#### Neovascularization

Neovascularization is a multiple process involving arteriogenesis, vasculogenesis, and angiogenesis [33]. ADSCs could secrete a range of different growth factors and miRNAs in therapeutic neovascularization. Under hypoxic conditions, ADSCs secrete VEGF, a critical angiogenic factor, at a significantly high level. The transplantation of preconditioned ADSCs increased the capillary density and restored cardiac function of ischemic hearts [31]. ADSC-derived miRNAs also play an important role in the process of neovascularization through promoting the proliferation and migration of ECs. Kang et al. reported that ADSCs promoted the migration of ECs by activating the miR-31/hypoxia-inducible factor-1 (HIF-1) pathway [34]. Furthermore, Togliatto et al. [35] reported that the reduced expression of miR-126 in obese ADSCs inhibited the extracellular signal-regulated protein kinase 1/2/mitogen-activated protein kinase (Erk1/2/MAPK) pathway in the cells, which consequently led to impaired angiogenesis. In addition, they indicated that overexpressed miR-126 could restore the angiogenic ability of obese ADSCs.

#### Anti-apoptosis

Myocardial infarction leads to a progressive loss of cardiomyocytes, resulting in left ventricular remodeling and congestive heart failure. The anti-apoptotic effect of ADSCs is executed by the secreted mRNAs and miRNAs. IGF-1 secreted by ADSCs protects cardiac cells by activating both the phosphatidylinositol-3-OH-kinase (PI3K) and MEK1 signaling pathway [36]. Furthermore, ADSC-secreted miRNAs were reported to salvage ischemic myocardium and downregulate the apoptosis process, leading to a significant improvement in cardiac function after myocardial infarction [37]. Lee et al. [38] reported that overexpressed miRNA-301a in human ADSCs inhibited apoptosis signal-regulating kinase 1 (ASK1) expression. ASK1 was previously shown to have an important role in CM death, and thus inhibition of ASK1 by miR-301a could promote the survival of injured CMs [38]. In addition, Salomone et al. indicated that ADSCs protected rats from acetaminophen-induced acute liver failure [39].

#### Anti-cardiac and vascular remodeling

Cardiac remodeling, characterized by cardiac hypertrophy and fibrosis, is a compensatory consequence of CVD and usually develops into heart failure. HGF is known as one of the main contributors to the anti-fibrosis function of ADSCs. In a preclinical swine model of myocardial ischemia and reperfusion injury, treatment with HGF attenuated cardiac hypertrophy, tissue fibrosis, and cardiac remodeling, and thus improved cardiac function [40].

Vascular remodeling is an adaptive reaction of the vessel to maintain constant flow. The progress involves hypertrophy, hyperplasia, and apoptosis of vascular cells as well as generation and degradation of extracellular matrix [41]. Liu et al. [42] observed that administration of exosomes from ADSCs significantly decreased intimal thickness in a mouse vein graft model. Moreover, this benefit effect was caused by decreased macrophage infiltration, attenuated inflammatory cytokine expression, and reduced activation of MAPK and PI3K signaling pathways [42].

### Engineered and pharmacologically modified ADSCs

**Fig. 3:**
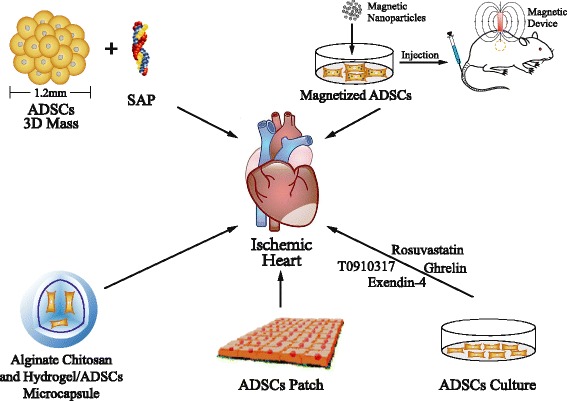
Engineered and pharmacologically modified adipose-derived stem cells (*ADSCs*). A summary of engineering and pharmacological strategies for improving survival and retention of transplanted ADSCs in ischemic hearts is shown, such as three-dimensional (*3D*) cultured ADSCs combined with self-assembling peptide (*SAP*), magnetic nanoparticle-loaded ADSCs, encapsulated ADSCs with chitosan and hydrogel, ADSC patch, and pharmacologically modified ADSCs

ADSCs have been shown to be an effective approach to ameliorating heart remodeling post-ischemia. The efficacy of stem cell therapies mostly depends on the survival and engraftment of the cells [43]. Different strategies have been introduced to promote their retention and engraftment after transplantation (Fig. 3).

#### Engineered ADSCs

Hamdi et al. [44] used scaffolds ensuring cell cohesiveness and co-delivery of implanted ADSCs; the engineered epicardial ADSC patches yielded less fibrosis (20.6% vs. 28.6%), more angiogenesis in the infarct area of myocardial infarction, and a significantly better restoration of cardiac function. Moreover, magnetically iron-labeled ADSCs are another effective approach to overcome this limitation [45]. ADSCs preloaded with superparamagnetic iron oxide nanoparticles were transplanted in infarct areas by intramyocardial injection. An externally applied static magnetic field significantly increased the number of localized “magnetic” cells, and thus markedly enhanced vascular density (374.49 ± 64.54 vessels/mm^2^ vs. 197.67 ± 60.70 vessels/mm^2^) and greatly decreased the percentage of apoptotic cardiomyocytes (0.86% ± 0.23% vs. 1.46% ± 0.46%) in the treated group compared with controls. This study also revealed that magnetic nanoparticle-loaded ADSCs enhanced heart function recovery after myocardial infarction, with the left ventricular ejection fraction (LVEF) elevated from 49.73% ± 6.85% to 58.87% ± 5.87% [45]. Microencapsulation of stem cells was used to increase the implanted ADSC retention in the infarcted myocardium. Paul et al. [46] and Follin et al. [47] encapsulated ADSCs with genipin-crosslinked alginate chitosan and cross-linked alginate hydrogel, respectively. The encapsulated ADSCs enhanced cell retention 3.5-fold, augmented vasculogenesis, and reduced fibrosis (infarct size: 21.6% ± 1.1% vs. 27.2% ± 3.1%) and cardiac dysfunction (fractional shortening: 24.2% ± 2.1% vs.19.1% ± 0.5%) compared to free ADSCs in a myocardial infarction model. A recent study reported an interesting method for ADSC transplantation. Three-dimensional cell masses (3DCMs), a type of spheroid ADSC mass, have demonstrated their therapeutic effects on various ischemic diseases via increasing cell retention [48, 49]. Kim et al. [50] combined 3DCMs with self-assembling peptides (SAPs) to further promote their curative effects. This combinatorial therapy presented better survival and angiogenesis of ADSCs than transplantation with 3DCMs alone [50].

#### Pharmacologically modified ADSCs

Pharmacological treatment of ADSCs, with rosuvastatin [51], ghrelin [52], T0901317 [53], exendin-4 [54], S-nitroso-*N*-acetyl-d,l-penicillamine (SNAP) [55], and NapFF-NO (FFGGG, and b-galactose caged nitric oxide) [56] etc., has increased the efficacy of ADSCs in promoting retention and the paracrine function of implanted cells. Rosuvastatin was used to promote the survival of ADSCs after transplantation into infarcted hearts. Zhang et al. [51] reported that rosuvastatin administration enhanced the viability and angiogenesis ability of ADSCs in a rat myocardial infarction model through the PI3K/Akt and MEK/ERK pathways [51]. Later, Han et al. found that ghrelin had an effect on ADSCs with a similar mechanism to rosuvastatin [52]. Wang et al. demonstrated that the liver X receptor (LXR) agonist T0901317 improved survival of ADSCs in infarcted hearts by modulating the TLR4/NF-κB and Keap-1/Nrf-2 signaling pathways [53]. Exendin-4 was reported to have the same beneficial effect as T0901317 on ADSCs [54]. Exendin-4 adjuvant therapy with ADCSs improved myocardial viability, and decreased oxidative stress and apoptosis by STAT3 activation through the phosphorylation of Akt and ERK1/2. In addition, exendin-4 could enhance the differentiation efficiency of ADSCs into CMs and VSMCs. This was associated with increased cardiac function and vascular density in the infarcted area [54]. Further research should be undertaken to elaborate the mechanism(s) of these protective effects.

### Clinical trials with ADSCs

Convincing evidence from ADSC transplantation studies on cardiovascular disorders have prompted several clinical applications. Although still in phase I/II, ADSC transplantation therapy is expected to be widely used in the treatment of CVDs in the coming years. Moreover, various completed and ongoing clinical trials have been conducted to investigate the safety, feasibility, and efficacy of the application of ADSCs in patients with cardiovascular disorders (Table 1).

**Table 1:** Completed and ongoing trials for heart disease using ADSCs

Clinicaltrials.gov identifier	Study design	Disease type	Route of delivery	End-point	Enrolled number	Status
NCT00442806 [5]	Parallel assignment, double-blind, phase I	Acute myocardial infarction	Intracoronary injection	Safety, cardiac function	48	Completed
NCT01502501	Single group assignment, open-label, phase I, phase II	Congestive heart failure	Intramyocardial/intravenous injection	6-minute walk test, LVEF, NYHA class	10	Recruiting
NCT02673164	Parallel assignment, double-blind, phase II	Heart failure	Intramyocardial injection	Safety, LVESV	138	Not recruiting
NCT00426868	Parallel assignment, double-blind, phase I	Ischemic myocardium	Intramyocardial injection	Safety, cardiac function	36	Completed
NCT01502514	Single group assignment, open-label, phase I, phase II	Congestive Heart Failure	Intramyocardial/intravenous injection	6-minute walk test, LVEF, NYHA class	10	Recruiting
NCT02387723	Single group assignment, open-label, phase I	Heart failure	Intramyocardial injection	Safety, LVESV, LVEF	10	Completed
NCT01449032	Parallel assignment, double-blind, phase II	Chronic heart failure	Intramyocardial injection	Exercise test	60	Active, not recruiting
NCT02052427 [6]	Parallel assignment, double-blind, phase II	Chronic myocardial ischemia	Intramyocardial injection	Change in Minnesota Living with Heart Failure Questionnaire	45	completed
NCT01556022 [6]	Parallel assignment, double-blind, phase II	Chronic myocardial ischemia	Intramyocardial injection	Safety, cardiac function	45	completed
NCT01709279	Single group assignment, open-label, phase I	Ischemic heart failure	Intracoronary	All cause harmful events	6	Recruiting
NCT01974128	Single group assignment, open-label, phase I, phase II	Acute myocardial infarction	Intramyocardial injection	Cardiac Improvement	10	Not recruiting
NCT01216995	Parallel assignment, double-blind, phase II	Acute myocardial infarction	Intracoronary injection	Reduction in infarct size, MACCE rates	216	completed

LVEF left ventricular ejection fracttion, LVESV left ventricular end-systolic volume, MACCE major adverse cardiac and cerebral events, NYHA New York Heart Association

The first study, the APOLLO Trial (NCT00442806) [5] is a randomized, double-blind, placebo-controlled clinical trial of ADSCs in the treatment of patients with ST-elevation acute myocardial infarction. This trial evaluated the safety and feasibility of ADSCs delivered via intracoronary injections in a dose of 20–40 million cells by major adverse cardiac and cerebral events (MACCE), magnetic resonance imaging (MRI), single-photon emission computed tomography (SPECT), and echocardiography. After 6 months of treatment, ADSCs promoted revascularization, improved the cardiac function, and reduced scar formation in the heart. The ATHENA trials I (NCT01556022) and II (NCT02052427) performed by Henry and colleagues focused on severe chronic myocardial infarction [6]. These trials are prospective trials with patients who are not eligible for percutaneous or surgical revascularization. The cells were delivered via an intramyocardial route. Results from the ATHENA trials were published recently; the processing and simultaneous delivery of ADSCs to patients with severe chronic ischemic cardiomyopathy was proved to elevate cardiac function and was accompanied by a remarkable improvement in the occluded blood perfusion. Kesten et al. carried out the ADVANCE study (NCT01216995), which involves 216 patients from 35 international clinical sites, aiming to investigate the efficacy of ADSC delivery on reducing infarct size. This trial is completed but its detailed data are not published yet.

All the above studies indicate that ADSC treatment could improve cardiac perfusion, reduce infarct size, and restore cardiac function after myocardial infarction. More importantly, all the completed clinical trials in Table 1 show a favorable safety profile and no tumor development with ADSC transplantation. However, Pendleton and colleagues found that ADSCs could develop into glioma in vitro [57]. In accordance with the study of Pendleton et al., Ra et al. [58] observed tumor development in nude mice 26 weeks after ADSC transplantation at the dose of 2 × 10^8^ cells/kg body weight, with no tumor induction at a lesser dose [58]. In addition, systematic ADSC application still faces some potential risks such as embolism and inflammation. In chronic cardiovascular diseases, the progressing apoptosis of cardiomyocytes and fibrosis play key roles in heart remodeling. The reversal of heart remodeling requires a lot of cardiomyocytes to replace the lost cells and fibrous tissue. However, due to the limited differentiation ability of ADSCs into cardiomyocytes, the cell transplantation may lead to a relatively poor therapeutic outcome. More clinical trials based on ADSC transplantation are expected in the near future.

## Conclusion

ADSCs are easily obtained and expanded, and are ideal candidates for cell-based therapy. The differentiation of ADSCs into CMs, ECs, and VSMCs has been induced by various reagents, growth factors, and genetic modification. In addition, ADSCs play an important role in anti-apoptosis, anti-cardiac remodeling, and angiogenesis through their paracrine effects. Despite the above regenerative characteristics, ADSCs face the major challenge of low survival and retention in the harsh ischemic microenvironment. Therefore, researchers have utilized engineering and pharmacological strategies to overcome theses difficulties. In animal models, ADSCs have been confirmed as an effective therapy for CVD. Treatment with ADSCs reduced infarct size and enhanced cardiac function through direct differentiation and paracrine effects. Phase I/II clinical trials have been conducted to test the safety and feasibility of ADSC therapy. Further phase III clinical trials are needed to achieve more reliable and efficient benefits. Although there are still many problems to solve, ADSC transplantation could be a promising means of treating cardiovascular disease in the near future.

## Abbreviations

3DCM: Three-dimensional cell mass
5-Aza: 5-Azacytidine
ADSC: Adipose-derived stem cell
ADVANCE: A Phase II Trial of Safety and Efficacy of ADRCs Delivered *Via* the Intracoronary Route in the Treatment of Patients With ST-elevation Acute Myocardial Infarction
AMI: Acute myocardial infarction
Ang II: Angiotensin II
APOLLO: A Randomized Clinical Trial of AdiPOse-derived Stem ceLLs in the Treatment of Patients With ST-elevation myOcardial Infarction
ASC: Adult stem cell
ASK1: Apoptosis signal-regulating kinase 1
ATHENA: Adipose-derived Regenerative Cells in the Treatment of Patients With Chronic Ischemic Heart Disease Not Amenable to Surgical or Interventional Revascularization
bFGF: Basic fibroblast growth factor
BM-MSC: Bone marrow-derived mesenchymal stem cell
CM: Cardiomyocyte
CVD: Cardiovascular disease
EC: Endothelial cell
ERK1/2: Extracellular signal-regulated protein kinase 1/2
ESC: Embryonic stem cell
HCN4: Hyper-polarization activated cyclic nucleotide gated potassium channel 4
HGF: Hepatocyte growth factor
HIF-1: Hypoxia-inducible factor-1
IGF-1: Insulin-like growth factor-1
IHD: Ischemic heart disease
LVEF: Left ventricular ejection fraction
LXR: Liver X receptor
MACCE: Major adverse cardiac and cerebral events
MAPK: Mitogen-activated protein kinase
MEK1: Mitogen-activated protein kinase 1
MRI: Magnetic resonance imaging
MSC: Mesenchymal stem cell
PI3K: Phosphatidylinositol-3-OH-kinase
SAN: Sinoatrial node
SAP: Self-assembling peptides
SM α-actin: Smooth muscle α-actin
SM-MHC: Smooth muscle myosin heavy chain
SPECT: Single-photon emission computed tomography
TGF: Transforming growth factor
VEGF: Vascular endothelial growth factor
VSMC: Vascular smooth muscle cell
